# Sulfadoxine-pyrimethamine parasitological efficacy against *Plasmodium falciparum* among pregnant women and molecular markers of resistance in Zambia: an observational cohort study

**DOI:** 10.1186/s12936-021-03596-3

**Published:** 2021-01-22

**Authors:** Enesia Banda Chaponda, Sungano Mharakurwa, Charles Michelo, Jane Bruce, Daniel Chandramoha, R. Matthew Chico

**Affiliations:** 1 Department of Biological Sciences, University of Zambia, Lusaka, Zambia; 2 Africa University, Fairview Road, Old Mutare, Mutare, Zimbabwe; 3 Department of Epidemiology, School of Public Health, University of Zambia, Lusaka, Zambia; 4 Strategic Centre for Health Systems Metrics and Evaluations, School of Public Health, University of Zambia, Lusaka, Zambia; 5 Department of Disease Control, Faculty of Infectious and Tropical Diseases, London School of Hygiene and Tropical Medicine, London, UK

**Keywords:** Intermittent preventive treatment of malaria in pregnancy (IPTp), Sulfadoxine-pyrimethamine (SP), DHPS double mutation (gly-437 + glu-540), DHFR triple mutation (asn-108 + ile-51 + arg-59), Quintuple mutation (DHFR triple + DHPS double), Sextuple mutation (DHFR triple + DHPS double + arg-581)

## Abstract

**Background:**

The World Health Organization recommends the provision of intermittent preventive treatment during pregnancy (IPTp) with sulfadoxine-pyrimethamine (SP) at 4-week intervals from gestational week 13 to delivery in areas of moderate to high malaria transmission intensity. However, the effect of IPTp-SP has been compromised in some areas due to parasite resistance, raising the importance of parasitological and chemoprophylactic surveillance, and monitoring SP-resistance markers in the *Plasmodium falciparum* population.

**Methods:**

Between November 2013 and April 2014 in Nchelenge, Zambia, 1086 pregnant women received IPTp-SP at antenatal-care bookings. Blood samples were collected on day 0, and on day 28 post-treatment to test for malaria parasites and to estimate SP parasitological efficacy in the treatment and prevention of parasitaemia. A random sample of 96, day 0 malaria-positive samples were analysed to estimate the prevalence of SP-resistance markers in the *P. falciparum* population.

**Results:**

The overall parasitological and prophylactic failure among women who had paired day 0 and day 28 blood slides was 18.6% (95% CI 15.5, 21.8; 109 of 590). Among pregnant women who had asymptomatic parasitaemia on day 0, the day 28 PCR-uncorrected parasitological failure was 30.0% (95% CI 23.7, 36.2; 62 of 207) and the day 28 PCR-corrected parasitological failure was 15.6% (95% CI: 10.6, 20.6; 32 of 205). Among women who tested negative at day 0, 12.3% (95% CI: 9.0, 15.6; 47 of 383) developed parasitaemia at day 28. Among the 96 malaria-positive samples assayed from day 0, 70.8% (95% CI: 60.8, 79.2) contained the DHPS double (Gly-437 + Glu-540) mutation and 92.7% (95% CI: 85.3, 96.5) had the DHFR triple (Asn-108 + Ile-51 + Arg-59) mutation. The quintuple mutation (DHFR triple + DHPS double) and the sextuple mutant (DHFR triple + DHPS double + Arg-581) were found among 68.8% (95% CI: 58.6, 77.3) and 9.4% (95% CI: 4.2, 16.0) of samples, respectively.

**Conclusion:**

The parasitological and chemoprophylactic failure of SP, and the prevalence of resistance markers in Nchelenge is alarmingly high. Alternative therapies are urgently needed to safeguard pregnant women against malarial infection.

## Background

Pregnant women in malaria-endemic areas are at high risk of *Plasmodium falciparum* infection and related consequences that include stillbirth [1, 2], small for gestational age [3, 4], preterm birth [4, 5], and low birthweight [4, 6]. Preterm birth and low birthweight are associated with marked increase in neonatal death [7–10]. The World Health Organization (WHO) recommends providing intermittent preventive treatment in pregnancy (IPTp) with sulfadoxine-pyrimethamine (SP) during antenatal care (ANC) contacts from the 13th gestational week until delivery at no < 4-week intervals in areas of moderate to high transmission intensity [11]. IPTp-SP is designed to improve birth outcomes by clearing parasitaemia at the time of dosing, and to prevent the effects of malarial infections acquired between antenatal contacts. However, the effectiveness of IPTp-SP has been undermined by malaria parasite resistance to SP [12, 13]. The WHO malaria treatment guidelines for the management of uncomplicated malaria state that countries should conduct routine surveillance of treatment efficacy and change the first-line therapy when parasite clearance rates decrease beneath 90% [14]. Even though IPTp-SP is given to asymptomatic pregnant women for the purposes of preventing the consequences of malarial infection during pregnancy, rather than for the treatment of symptomatic and uncomplicated malaria, surveillance is needed to determine when and where alternative therapies and interventions may be required. This study quantified the therapeutic and chemoprophylactic efficacy of IPTp-SP and the proportion of malaria parasites containing mutations in the dihydropteroate synthase (DHPS) and dihydrofolate reductase (DHFR) genes associated with resistance to SP [15–21].

This observational cohort study was conducted in Nchelenge, a rural northern district in the Luapula Province with a population of 173,680 [22]. The objectives of the study were to estimate: (1) the in vivo parasite-clearance efficacy and prophylactic effects of SP in pregnant women during a 28-day period and, (2) the prevalence of DHFR and DHPS mutations associated with SP resistance in the same population. Nchelenge has a tropical climate and rainy season from November to April, followed by a dry season between May and October. Despite the seasonality of precipitation, malaria transmission occurs year-round due to the district’s proximity to Lake Mweru and surrounding wetlands.

## Methods

Details of enrolment procedures including inclusion and exclusion criteria have been previously reported [23]. Briefly, 1086 consenting women of all ages and gestational-ages < 32 weeks were enrolled consecutively at ANC booking if they provided consent, self-reported not having taken anti-malarial and/or antibacterial drugs in the previous month, and were willing to have their HIV test results from routine testing recorded by study staff. Participants provided peripheral blood samples by finger prick at enrolment (day 0) for preparation of two blood smears and four blood spots on circles of Whatman^®^ filter paper. Thick smear-slides and dried blood spots were, respectively used to diagnose malaria by microscopy and polymerase chain reaction (PCR). Participants received directly observed IPTp-SP during the same consultation. The follow up visit was conducted on day 28 post-treatment during which peripheral blood was collected for malaria diagnosis by microscopy and PCR. Participants verbally confirmed having not taken any anti-malarial treatment between day 0 and day 28.

In alignment with the WHO treatment efficacy protocol [24], sample size was determined using classical statistical methods. It was assumed that the proportion of parasitological failures in the study population was 10%. To detect 10% parasitological failure with 95% confidence level and 5% precision, a sample size of 138 asymptomatic pregnant women was required. Only women who were eligible to receive IPTp-SP were included. Study staff visited the homes of participants on day 28 if they did not present at the facility. Participants who developed symptoms of malarial infection before day 28 were tested and, if positive, were given rescue treatment by the health centre staff in accordance with national guidelines.

### Malaria microscopy, DNA extraction and PCR amplification

Laboratory staff stained thick blood films using 10% Giemsa which were then read by two independent microscopists. Details of slide-reading methods have been reported elsewhere [23]. Parasite DNA extraction from dried blood spots was carried out using the Chelex method as presented previously [25]. The detection of *P. falciparum* was conducted using a nested-PCR method as described by Snounou et al. [26] with modifications to the PCR parameters [27].

### Detection of SP resistance markers

A sample size of 96 was needed to detect a 50% prevalence of quintuple mutation among pregnant women with 95% CIs ± 10%. Parasite DNA template extracted for malaria detection by PCR was used for this part of the study. Nested PCR and restriction enzyme digestion methods were both used to detect antifolate drug resistance polymorphisms in the DHFR and DHPS genes [28]. Briefly, all PCR reactions were carried out in total volumes of 25 µl using Thermo Scientific^®^ Dream Taq PCR Master Mix (2X) and 0.5 µM of each primer and 2 µl of template. A 2 µl of the primary amplicon was used as a template in the secondary reaction. Negative and positive controls were included in every batch of sample processing, from extraction to amplification, and finally electrophoresis. Secondary PCR amplicons were analysed by gel electrophoresis to confirm amplification and band intensity before enzyme digestion. Restriction-digest assays were set up following manufacturer instructions; 4 µl of amplicon was used as substrate in the reaction mix. For samples showing faint bands in the nested PCR product, 6–8 µl was used as substrate. Amplicon and restriction fragments were analysed on ethidium bromide 2% agarose gels and visualized under ultraviolet transillumination on a Biosens (Genescope V1.76) digital imaging system.

### Determination of in vivo parasite-clearance efficacy and prophylactic effect of SP

In vivo parasite-clearance efficacy and prophylactic effect of SP was established based on malaria microscopy [24] and merozoite surface protein-2 (MSP2) genotyping [29, 30] which differentiated cases of recrudescence from reinfection. The two blood smears collected at enrolment on day 0 and day 28 post-treatment were used to estimate the in vivo parasite-clearance efficacy and prophylactic effect. Women were classified as having parasitological-clearance failures if they had a malaria-positive blood smear both at day 0 and day 28, and genotyping of MSP2 confirmed recrudescence and ruled out new infections [29, 30]. Women who had a negative blood slide at day 0 and became slide-positive at day 28 and those who were positive at day 0 and had new infections by day 28 defined by MSP2 genotyping, were classified as prophylactic failures.

Polymorphic regions of MSP2 were amplified by nested PCR. Primary PCR primers corresponding to the conserved sequence flanking this region [31] were used, whereas the secondary PCR primers were used to amplify the IC3D7 and FC27 allelic families of MSP2 [32]. For controls, DNA from HB3 and 3D7 laboratory strains were used. Briefly, all PCR reactions were carried out in total volumes of 25 µl using Thermo Scientific^®^ Dream Taq PCR Master Mix (2X) and 0.5 µM of each primer and 2 µl of template. A 2 µl of the primary amplicon was used as a template in the secondary reaction.

The secondary amplicon from each sample was then analysed using electrophoresis on 2% ethidium bromide stained agarose gels. Samples from individual participants were loaded in adjacent lanes. In cases where there was no amplification, PCR was repeated using five-times the quantity of template DNA. In cases where no amplicon was detected after the second reaction, amplification was classified unsuccessful [33]. Two independent laboratory personnel compared band sizes manually; any discordant classification was settled by a third laboratory staff member. Reinfection and recrudescence were defined as described elsewhere [33]. Briefly, reinfection was defined as having completely different alleles between parasites from day 0 and from day 28; recrudescent infection was assigned if the same allele was found between parasites at day 0 and day 28.

### Statistical analysis

Data were double-entered in EpiData version 3.1 software [34], cleaned, processed and analysed using Stata software version 13 [35]. This involved visual checks for consistency and validity, as well as variable frequencies to check for missing data. Variables were then recoded and composite variables generated for the DHFR triple (Ile-51 + Arg-59 + Asn-108), DHPS double mutant (Gly-437 + Glu-540), quintuple (DHFR triple + DHPS double) and sextuple (quintuple + Gly-581) mutants.

Parasitological failure was defined as presence of parasites on day 0 and day 28 post treatment that was deemed to be recrudescence and not a new infection. Prophylactic failure was defined as the presence of parasitaemia in previously aparasitaemic women on day 28 post-treatment, as well as parasitaemia deemed to be a new-infections in women who tested positive at day 0 and day 28. Overall parasitological and prophylactic failure was defined as the inability of SP to clear existing parasites and to prevent infection within 28 days of administration.

Baseline characteristics among asymptomatic parasitaemic women were compared between paucigravidae (primi- and secundigravidae combined) and multigravidae using appropriate tests, namely Chi-squared test for proportions, t-tests for means and Mann Whitney for medians. Prevalence estimates of the DHFR triple, DHPS double; DHFR + DHPS quintuple and sextuple mutants and their 95% CIs were then calculated.

Parasitological and prophylactic failure in paucigravidae and multigravidae women was compared using Chi-squared test, while the median age of women who experienced failure compared to those who did not was assessed with the Mann Whitney test.

## Results

**Fig. 1:**
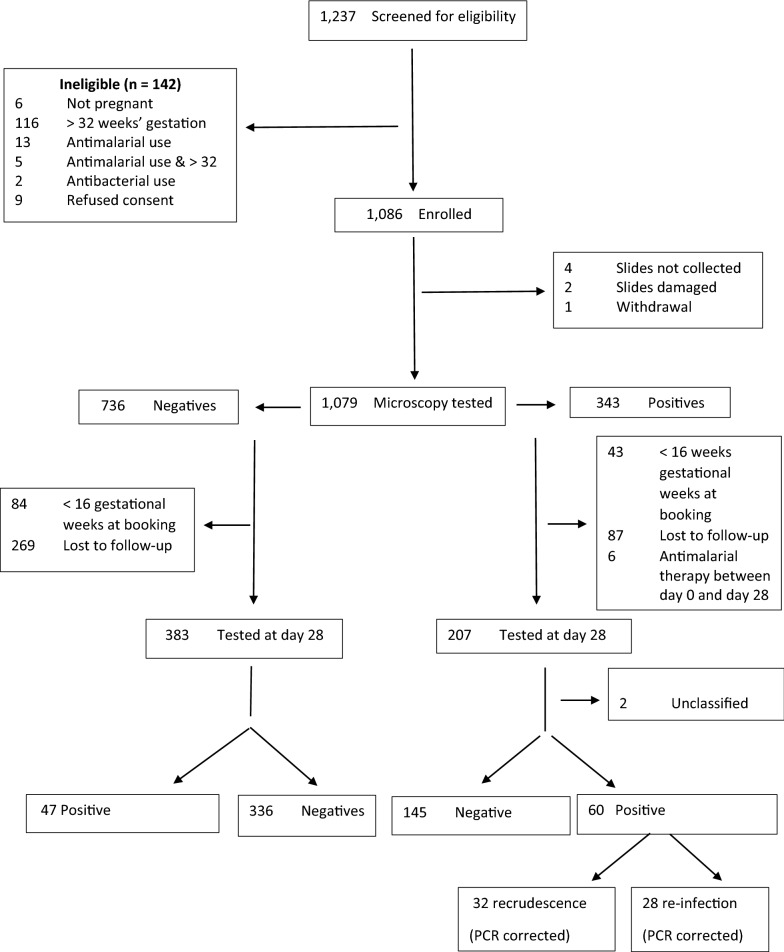
Enrolment flow diagram for in vivo efficacy and prophylactic effect analyses of sulphadoxine-pyrimethamine given to pregnant women as IPTp in Nchelenge District, Zambia

A total of 1086 ANC attendees were enrolled from November 2013 to April 2014. Among them, 343 women had asymptomatic parasitaemia at day 0 (Fig. 1). Of these, 56.3% (n = 193) and 43.7% (n = 150) were paucigravidae and multigravidae, respectively. The median age of paucigravidae was significantly lower than that of multigravidae, 18.4 versus 27.3 years, *P* < 0.001. Use of bed nets on the previous night was significantly higher among multigravidae than paucigravidae, respectively, 42.7% versus 27.5%, *P* = 0.003. Parasite count measured by geometric mean was lower in multigravidae compared to paucigravidae, 849 versus 1310, *P* = 0.001. Other characteristics of asymptomatic parasitaemic women in this cohort stratified by gravidity are shown in Table 1.

**Table 1:** Baseline characteristics

Characteristic	Total (N = 343)	Paucigravidae (N = 193)	Multigravidae (N = 150)	*P*-value*
Age, median (interquartile range)	22 (19–28)	19 (18–21)	28 (25–32)	< 0.001
Age ≤ 20 years, %	43.7%	70%	10%	< 0.001
Used a bed net last night, %	34.1%	27.5%	42.7%	0.003
Maternal weight in kilograms, mean (SD)	52.6 (8.7)	50.8 (7.0)	55.0 (10.0)	< 0.001
Education in years, mean (SD)	7.6 (7.6)	8.2(7.1)	6.8 (8.2)	0.079
Gestational age in weeks at enrolment, mean (SD)	21.6 (4.6)	20.9 (4.3)	22.6 (4.6)	0.001
Parasite density, geometric mean (95% CI)	1082 (962–1217)	1310 (1120–1532)	849 (713–1010)	0.001

Asymptomatic pregnant women of Nchelenge District, Zambia, enrolled in a modified in vivo efficacy study of sulphadoxine-pyrimethamine stratified by gravidity

*SD* = standard deviation

*P-value comparing paucigravidae and multigravidae, Chi-squared for proportions, t-test for means, Mann–Whitney for medians

Of the women who attended the day 28 visit among those who received IPTp-SP at day 0 and were eligible to attend the day 28 visit, 37.6% (356 of 946) were lost to follow-up. The proportions of socio-demographic factors such as age, gravidity and marital status, did not differ between the lost to follow-up group and those who were retained with the exception of the number of years of schooling. Among women who were lost to follow-up, 54.4% reported spending 7 years and above in school versus 65.5% among those who were retained at day 28, *P* < 0.001.

### Submicroscopic malaria infection

The prevalence of malaria detected by polymerase chain reaction and microscopy have been reported elsewhere and were 57.8% (621 of 1074) and 31.8% (343 of 1079), respectively. The prevalence of submicroscopic malaria infection (microscopy negative but PCR positive) was 38.9% (285 of 731).

### Parasitological-clearance efficacy of IPTp-SP over a 28-day period

Among women who tested positive for malaria on day 0 measured by microscopy, 60.3% (207 of 343) were tested again at day 28 post-treatment. Sulfadoxine-pyrimethamine cleared parasitaemia present on day 0 in 70.0% (145 of 207) of pregnant women confirmed by day 28 test (Table 2). Among the 62 women who had parasitaemia on both day 0 and day 28, MSP-2 genotyping was unsuccessful in 2 women, 32 were classified as recrudescent infections and 28 were re-infections. Thus, the day 28 PCR-corrected parasitological failure was 15.6% (95% CI: 11.2, 21.3; 32 of 205).

**Table 2:** Estimates of treatment and prophylactic failure of sulphadoxine-pyrimethamine among antenatal care attendees in Nchelenge District, Zambia

Category	Number of women	Proportion of parasite-positive women at day 28, % (n)	95% Confidence intervals
Overall parasitological and prophylactic failure^a^	590	18.6 (109)	15.5, 21.8
Total prophylactic failure^b^	411	18.3 (75)	14.8, 22.3
Day 0 malaria positives			
Day 28 PCR uncorrected parasitological failure	207	30.0 (62)	23.7, 36.2
Day 28 PCR corrected parasitological failure	205^c^	15.6 (32)	11.2, 21.3
Day 28 PCR corrected reinfection	60	46.7 (28)	34.2, 59.6
Day 0 malaria negatives			
Day 28 PCR uncorrected prophylactic failure	383	12.3 (47)	9.0, 15.6

*PCR* polymerase chain reaction diagnostic methods

^a^All positives among women who took SP at enrolment and tested malaria positive at day 28 which includes prophylactic and parasitological failures

^b^The total includes aparasitaemic at day 0 and parasitaemic at day 28 plus the 28 women who were parasitaemic at day 0 and day 28 due to reinfection

^c^Two samples could not be differentiated between recrudescence or reinfection, which is reflected in the smaller denominator (205) of samples after PCR correction for parasitological failure

Among women with a negative malaria smear at day 0, 52.0% (383 of 736) were also screened at day 28. Of these women, 12.3% (47 of 383) became malaria parasite positive at day 28 post-treatment (Table 2). In addition, of the 62 women who had malaria parasitaemia at day 0 and day 28 post treatment, 28 were categorized as reinfections. Thus, the total prophylactic failure was 18.3% (95% CI: 14.8, 22.3; 75 of 411). Overall parasitological and prophylactic failure by day 28 post IPTp-SP administration was 18.6% (95% CI: 15.5, 21.8; 109 of 590) (Table 2).

The proportion of parasitological failure observed among paucigravidae was much higher than that observed among multigravidae, 22.4% (26 of 116) versus 6.7% (6 of 89), *P* = 0.002. Similarly, the proportion of prophylactic failure was higher among paucigravidae, 28.4% (38 of 134) than multigravidae13.4% (37 of 277), *P* < 0.001. Younger women were more likely to experience parasitological failure. The median age of women who experienced parasitological failure was 19 years (IQR, 17.5, 21.5) compared to 22 years among those whose parasitaemia cleared (IQR, 19, 28), *P* < 0.001. The median age of women who experienced prophylactic failure was 24 (IQR, 20–29) years while that of women who maintained their malaria negative status was 26 (IQR, 22–32) years*, P* = 0.027.

### Prevalence of DHFR and DHPS mutations associated with SP resistance

**Fig. 2:**
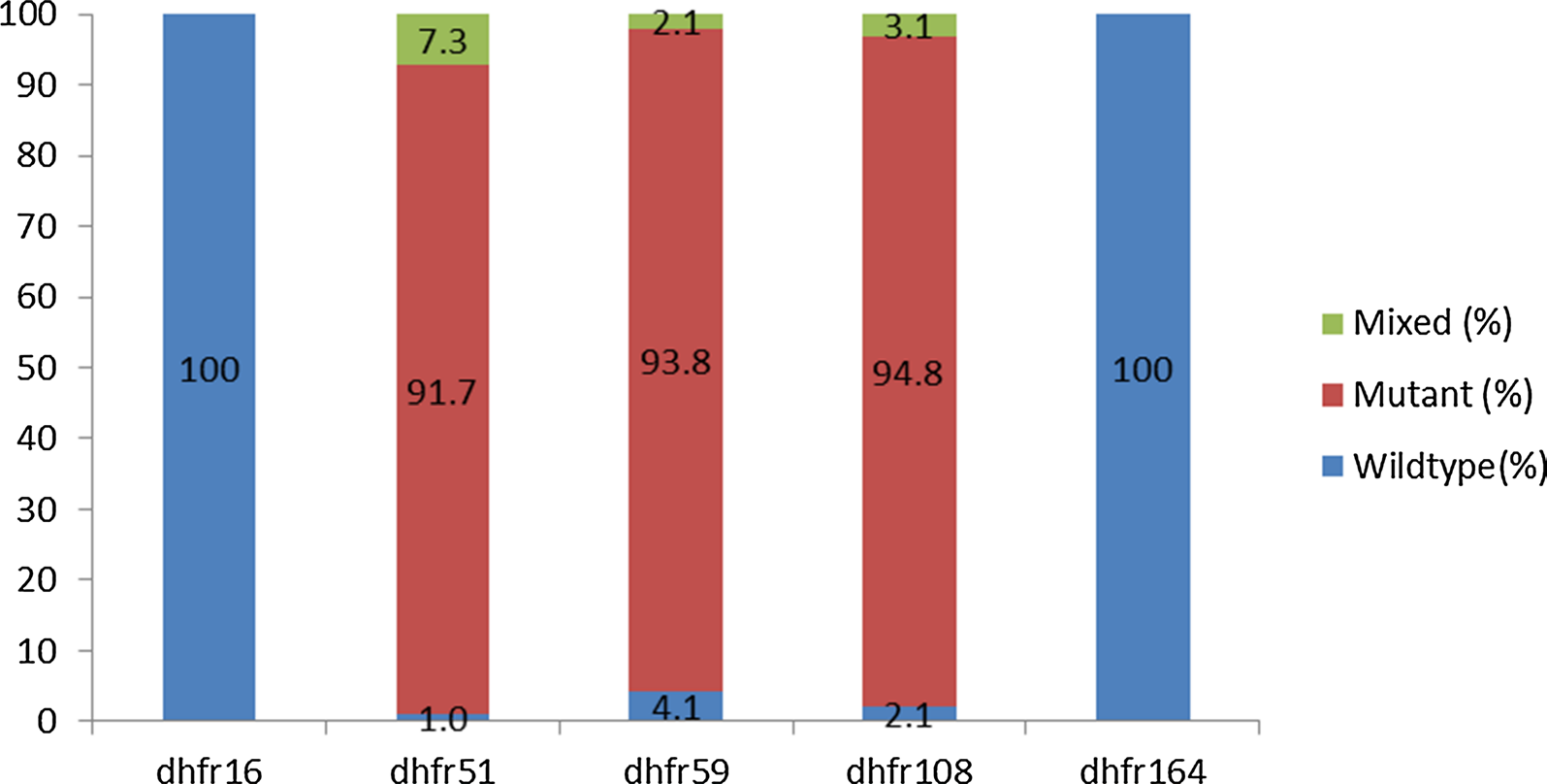
Prevalence of *Plasmodium falciparum* DHFR mutations in pregnant women of Nchelenge District, Zambia

**Fig. 3:**
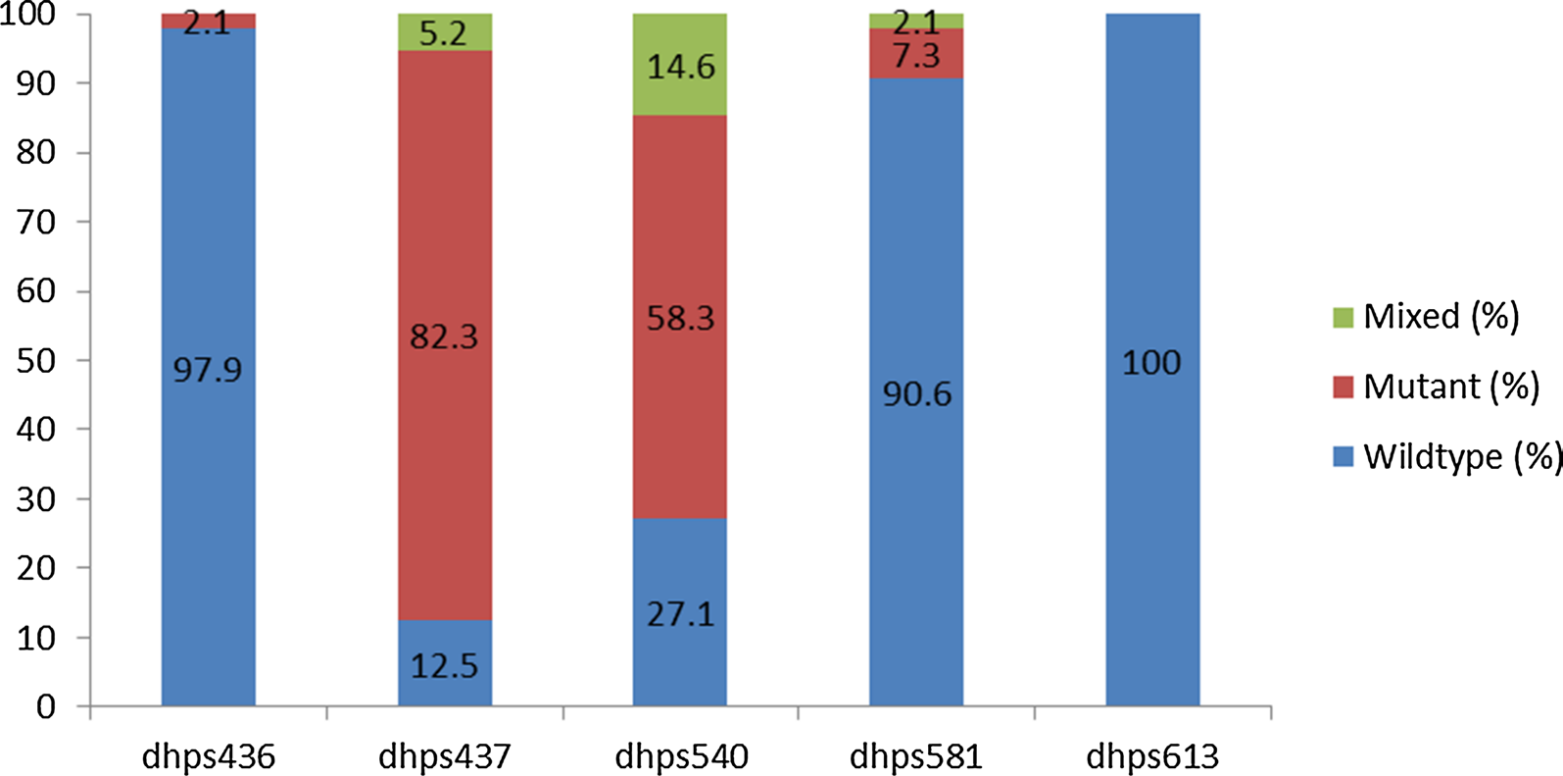
Prevalence of *Plasmodium falciparum* DHPS point mutations in pregnant women of Nchelenge District, Zambia

There was near saturation of the pyrimethamine-resistant DHFR Asn-108 mutation at 94.8% (91 of 96) among the *P. falciparum* positive samples. Mixed infection of Asn-108 occurring with the wild type Ser-108 was observed in 3.1% (3 of 96) of samples. High levels of the DHFR Ile-51 and Arg-59 mutants as shown in Fig. 2 as well as the DHPS Gly-437 and Glu-540 in Fig. 3. Table 3 contains the prevalence of the double, triple, quintuple and sextuple mutants. Proportions of specific mutants were found as follows: 70.8% (68 of 96) had the DHPS double mutant (Gly-437 + Glu-540); 92.7% (89 of 96) had the DHFR triple mutant (Asn-108 + Ile-51 + Arg-59); 68.8% (66 of 96) had the quintuple mutant (DHFR triple + DHPS double). The mutation most associated with SP treatment failure in East Africa, the sextuple mutant (DHFR triple + DHPS double + Arg-581), was observed in 9.4% (9 of 96) of samples with 2 of these occurring as mixed infections with the wild type. Table 4 summarizes the number of samples that carried a particular combination of point mutations associated with SP resistance.

**Table 3:** *Plasmodium falciparum* antifolate multiple mutants among 96 malaria-positive pregnant women at antenatal care booking in Nchelenge District, Zambia

Mutant	Proportion of women with parasites containing mutations (n)	95% Confidence intervals
DHFR triple	92.7 (89)	85.3, 96.5
DHPS double	70.8 (68)	60.8, 79.2
DHFR/DHPS quintuple	68.8 (66)	58.6, 77.3
DHFR/DHPS sextuple	9.4 (9)	4.2, 16.0

**Table 4:** Results of DHFR and DHPS genotyping of *Plasmodium falciparum* samples from 96 pregnant women at antenatal care booking in Nchelenge District, Zambia

Number of samples	dhfr51	dhfr59	dhfr108	dhps437	dhps540	dhps581
1	−	−	−	−	−	−
2	+	−	+	−	−	−
2	+	+	+	−	+	−
1	+	−	+	+	+	−
1	+	+	−	+	+	−
7	+	+	+	−	−	−
16	+	+	+	+	−	−
57	+	+	+	+	+	−
9	+	+	+	+	+	+

− = wild type

+  = Mutant or mixed genotype

## Discussion

Overall, almost one-fifth (18.6%) of pregnant women in the study experienced a parasitological and prophylactic failure of SP at day 28 post-IPTp treatment in an area where 68.8% of malaria parasites carried the quintuple mutant and 9.4% expressed the sextuple mutant. Sulfadoxine-pyrimethamine retained partial efficacy in parasite clearance among pregnant women who had asymptomatic malarial infections. Similar observations have been made elsewhere despite a moderate to high prevalence of the quintuple mutant and presence of the sextuple mutant [36, 37].

The risk of parasitological failure was higher among paucigravidae than multigravidae. This may be partially attributable to primigravidae and secundigravidae having acquired less semi-immunity to malarial infection in pregnancy than multigravidae [3, 38, 39] and, therefore, relying more on drug-action to clear parasites. There was a difference, as well, among gravidae and use of insecticide-treated bed nets where a higher proportion was observed among multigravidae compared to paucigravidae.

It is difficult to establish the precise prophylactic failure rate because it is unknown how many women who tested negative by microscopy at day 0 may have later been exposed to malarial infection post-treatment. This is because some women may have become infected after receiving IPTp-SP, cleared the parasitaemia to reflect important prophylactic effect but were indistinguishable from other women who may have never have been exposed to the parasite. Nonetheless, calculating the prophylactic failure based on the number of malaria positive women from those who tested negative at day 0 is a reasonable and common proxy for the estimate of true prophylactic failure, especially in areas with moderate to high malaria transmission like this study area [40].

Only MSP-2 genotyping was conducted to distinguish recrudescence from reinfection in the current study. Although genotyping for genes MSP-1, MSP-2 and GLURP was not done in this study, MSP-2 results alone are reliable since the gene is known to provide a more accurate measure of treatment outcomes compared to MSP-1 and GLURP [33].

Point mutations associated with SP resistance were very common in this study. The prevalence of both the quintuple and the sextuple mutants among pregnant women was moderate but higher than recorded in earlier studies, both of which were conducted in the same province, Luapula [37]. The first of these prior studies documented the sextuple mutant among pregnant women at two health centres in Mansa District, 250 kms from the Nchelenge District between January 2010 and May 2011. The prevalence of the quintuple and sextuple mutation was 63.0% and 2.0%, respectively [37]. The second study carried out between February and April of 2013 among pregnant women in the Nchelenge District reported the prevalence of the quintuple and sextuple mutant to be 17.0% and 3.0%, respectively [41]. The current findings suggest the quintuple mutation is as common in Nchelenge as it is Mansa with the sextuple mutant on the rise in Nchelenge. This is cause for concern. The addition of the 581G to form the sextuple mutation renders malaria parasites ‘super resistant’ to SP and is strongly associated with treatment failure among cases of uncomplicated malarial infection [42]. Nchelenge appears to be on a related threshold based on a meta-analysis that found 2 or more doses of IPTp-SP no longer protected against the incidence of low birthweight among multigravidae where the prevalence of 581G was > 10.1% [43].

Only four studies conducted in Zambia have estimated the prevalence of SP resistance markers, two in the general population [44, 45] and two in pregnant women [37, 41]. Monitoring the prevalence of SP-resistance markers and the in vivo efficacy of SP in Zambia is critically important, especially in areas of high malaria transmission. Although IPTp-SP is not given to patients with uncomplicated malaria, surveillance is critically important for guiding policymakers around decisions related to when and where alternative therapies and interventions might be required. However, part of the challenge with IPTp and resistance monitoring is that there are no clear second-line therapies to promote. The leading candidate to replace SP is dihydroartemisinin-piperaquine (DP) which is superior to SP in terms of reducing malarial infections among pregnant women. However, a recent meta-analysis of pooled data from three IPTp trials suggests that DP is not superior to SP in terms of reducing the incidence of low birthweight [46]. Moreover, a mediation analysis using the same trial data showed that protection conferred by SP against low birthweight is actually derived more from its non-malarial properties than its anti-malarial effects [46]. Consequently, to achieve superiority, DP needs to have a partner compound that confers non-malarial protection against low birthweight. Alternatively, SP could be combined with a potent anti-malarial therapy, perhaps even DP, to improve its anti-malarial protection while maintaining its non-malarial effects against low birthweight. Clinical trials are urgently needed to identify a clear path forward.

The prevalence of submicroscopic malaria infection among pregnant women in the current study was considerable. Other studies have found wide spread submicroscopic malaria infections [47–49]. Many asymptomatic infections are submicroscopic and can only be detected by molecular methods. Achieving malaria elimination requires targeting the human reservoir of infection, including those with asymptomatic infection [50].

The limitations in this study include the fact that samples were only collected at day 0 and day 28. These collection time points do not allow for determining early parasitological failure. Secondly, samples were not collected from the six women who verbally indicated that they had taken anti-malarial drugs before day 28, therefore requiring their exclusion from treatment-failure analysis at day 28. Another limitation is the fact that some of the women who tested negative on day 0 could have had submicroscopic infections which became positive by day 28 which may have affected the estimate of prophylactic failure.

The number of women who were lost to follow-up was large. However, baseline characteristics did not differ between women lost to follow-up and those who were followed to day 28, with one exception: the number of years of schooling. This could have affected the results in terms of proportions of parasite clearance and parasitological failure if women lost to follow-up are different from those who were retained at day 28. However, given that these women are residents of the same geographic area where high malaria endemicity affects the community broadly [40], it is difficult to know whether this difference in years of schooling would have changed this observations.

## Conclusions

The quintuple and sextuple mutants were observed in this study population. Sulfadoxine-pyrimethamine retains partial efficacy in clearing parasites in pregnant women with moderate prevalence of the highly resistant quintuple mutation. These results suggest that continued monitoring is essential for future policy-making and there is a clear need to identify alternative regimens for use in IPTp.

## Data Availability

The datasets used and/or analysed during the current study are available from the corresponding author upon reasonable request.
